# Supplementary material for: The Effect of Pleural Effusion on Prognosis in Patients with Non-Small Cell Lung Cancer Undergoing Immunochemotherapy: A Retrospective Observational Study
Source: Cancers (Basel). 2022 Dec 14;14(24):6184. doi: 10.3390/cancers14246184 (PMC9776517; doi:10.3390/cancers14246184)
Supplement: Supplementary file 1 [file cancers-14-06184-s001.zip › cancers-2042009-supplementary.pdf]

Table S1. Characteristics of the patients with PE.

	Without bevacizumab	With bevacizumab	
	n = 99	n = 22	p-value
Age (years)			
median (range)	70 (42-84)	70 (51-82)	
≥ 70	55 (55.6%)	12 (54.6%)	1.00
< 70	44 (44.4%)	10 (45.4%)	
Sex			
female	16 (16.1%)	3 (13.6%)	1.00
male	83 (83.9%)	19 (86.4%)	
Performance status			
0-1	86 (86.9%)	19 (86.4%)	1.00
≥ 2	13 (13.1%)	3 (13.6%)	
Smoking history			
never	14 (14.1%)	2 (9.0%)	0.734
current or former	85 (85.9%)	20 (91.0%)	
Histology			
squamous	24 (24.2%)	1 (4.5%)	0.043
non-squamous	75 (75.8%)	21 (95.5%)	
PD-L1 expression			
< 1%	33 (33.4%)	5 (22.8%)	0.220
1-49%	28 (28.3%)	7 (31.9%)	
≥ 50%	20 (20.1%)	2 (9.0%)	
Unknown	18 (18.2%)	8 (36.3%)	
EGFR or *ALK* gene alteration	3 (3.0%)	6 (27.2%)	<0.001
Disease stage			
advanced	83 (83.9%)	15 (68.2%)	0.130
rec	16 (16.1%)	7 (31.8%)	
ICI drug			
Pembrolizumab	95 (96.0%)	0 (0.0%)	<0.001
Atezolizumab	4 (4.0%)	22 (100.0%)	

PE: pleural effusion, PD-L1: programmed death-ligand 1, EGFR: epidermal growth factor receptor, ALK: anaplastic lymphoma kinase, rec: postoperative or postchemoradiotherapy recurrence, ICI: immune checkpoint inhibitor.

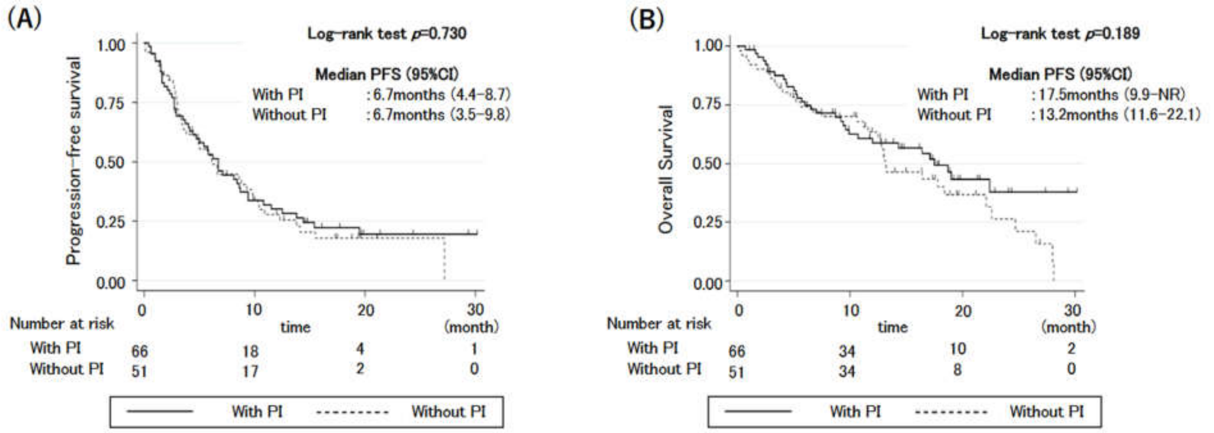

Figure S1. Kaplan-Meier curves for (A) progression-free survival and (B) overall survival in patients with prior pleural intervention (PI) and those without it.
